# Innovative anti-proliferative effect of the antiviral favipiravir against MCF-7 breast cancer cells using green nanoemulsion and eco-friendly assessment tools

**DOI:** 10.1038/s41598-024-78422-2

**Published:** 2024-11-14

**Authors:** Eman Abd-Elrasheed, Sally A. Fahim, Christine K. Nessim, Sara Nageeb El-Helaly

**Affiliations:** 1 Department of Pharmaceutics and Industrial pharmacy, Pharmacy Program, St. Petersburg University, Cairo, Egypt; 2 Department of Biochemistry, School of Pharmacy, Newgiza University (NGU), Newgiza, km 22 Cairo-Alexandria Desert Road, Giza, 12577 Egypt; 3 Department of Pharmaceutical Chemistry, Faculty of Pharmacy, Ahram Canadian University, 6th October City, Cairo, Egypt; 4 Department of Pharmaceutics and Industrial Pharmacy, Faculty of Pharmacy, Cairo University, Cairo, Egypt

**Keywords:** Favipiravir, Green nanoemulsion, Cytotoxicity, Breast cancer, Greenness assessment, Breast cancer, Cancer therapy, Pharmaceutics

## Abstract

Telomerase enzyme prevents telomere shortening during division, having human telomerase reverse transcriptase (hTERT) as its catalytic subunit. Favipiravir (FAV), an RNA-dependent RNA polymerases inhibitor, shared structural similarity with hTERT and thus assumed to have cytotoxic effect on cancer cells, in addition to its prophylactic effect to immunocompromised cancer patients. Nanoemulsion (NE) is a potential tumor cells targeting delivery system, thereby enhancing therapeutic efficacy at the intended site, mitigating systemic toxicity, and overcoming multidrug resistance. The objective of this study is to develop a green FAV nanoemulsion (FNE) that is environmentally friendly and safe for patients, while aiming to enhance its cytotoxic effects. The study also highlights the environmental sustainability of the developed RP-HPLC method and assesses its greenness impact. The FNE formulation underwent thermodynamic stability testing and *invitro* characterization. Greenness was assessed using advanced selected tools like the Analytical Eco-Scale (AES), Analytical Greenness Metric for Sample Preparation (AGREEprep), and green analytical procedure index (GAPI). The cytotoxic potential of FNE was screened against MCF-7 breast cancer and Vero normal cell lines using SRB assay. Stable and ecofriendly FNE was formulated having a particle size (PS) of 25.29 ± 0.57 nm and a zeta potential of -6.79 ± 5.52 mV. The cytotoxic effect of FNE on MCF-7 cells was more potent than FAV with lower IC50 while FNE showed non-toxic effect on VERO normal cell line. Therefore, the FAV nanoemulsion formulation showed targeted cytotoxicity on MCF-7 cells while being non-toxic on normal Vero cells.

**Supplementary Information:**

The online version contains supplementary material available at 10.1038/s41598-024-78422-2.

## Introduction

Breast cancer represents the foremost type of malignancy affecting women worldwide, with a high incidence and mortality caused among malignant tumors^1^. In 2020, breast cancer accounted for over 2.3 million newly diagnosed cancer cases and 685,000 mortalities. It is projected that by 2040, the worldwide burden of breast cancer will continue to grow, with an estimated three million new cases and one million deaths per year stemming solely from population growth and aging^2^. Despite significant advancements in the detection and treatment of breast cancer, the adverse effects associated with systemic chemotherapy remain a considerable concern^3,4^. Our objective is to explore the potential of reformulating a repurposed drug to exhibit fewer adverse effects for breast cancer treatment.

The preservation of telomere length is associated with cellular immortality. Telomerase, a ribonucleoprotein enzyme complex, plays a crucial role in preventing telomere shortening during cell division cycles. This complex includes the catalytic subunit known as human telomerase reverse transcriptase (hTERT)^5^. Reports indicate that elevated levels of telomerase and hTERT have been observed in more than 85% of human carcinomas. These elevated levels have shown a correlation with increased resistance to treatment^5–8^. Accordingly, telomerase and hTERT inhibitors have demonstrated potential effective shrinkage of breast cancer cells by impeding the proliferation of tumor cells^9–12^. Even though hTERT is being classified as an RNA-dependent DNA polymerase, recent analysis of its three-dimensional structure has unveiled its structural resemblance to viral RNA-dependent RNA polymerases (RdRps). This includes the characteristic right-handed architecture. Based on these findings, we suggest that inhibitors targeting RdRps could potentially exhibit an anti-tumor effect^12,13^.

**Fig. 1:**
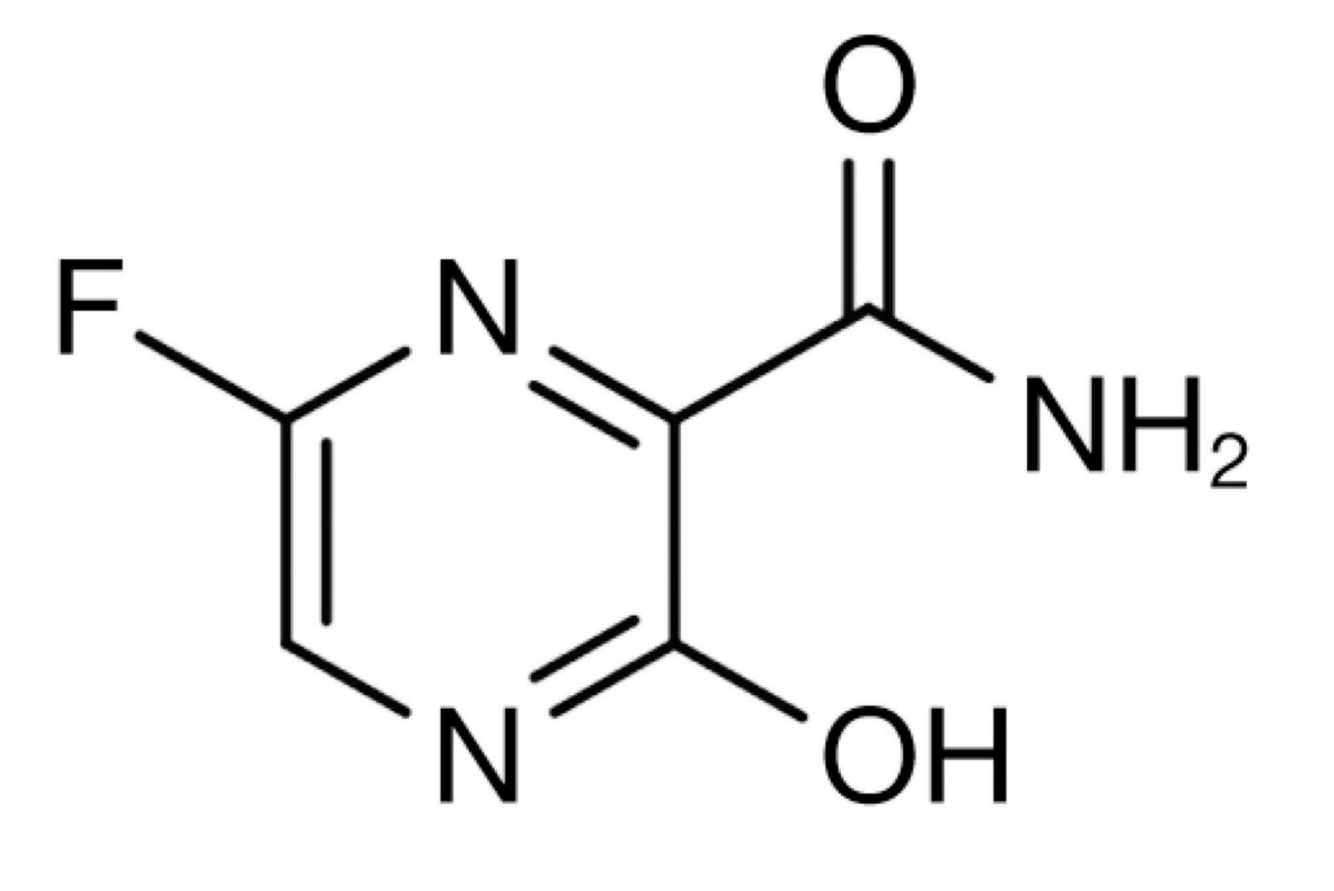
Chemical structure of Favipiravir (FAV).

The antiviral drug favipiravir (FAV) is a 6-Fluor-3-oxo-3,4-dihydro-2-pyrazincarboxamid as shown in Fig. 1. It is excreted mainly in the urine after being metabolized by aldehyde and xanthine oxidases^14^. It is a purine nucleic acid analog which act as an inhibitor of RdRp and gained significant attention worldwide as an emergency-approved treatment for COVID-19 in several countries, including Japan, Italy, Russia, Turkey, and Egypt^15,16^. FAV was reported to effectively treat COVID-19, as it promotes only within 7 days the viral clearance with reported clinical improvement within 14 days. Furthermore, FAV has demonstrated efficacy against various RNA viruses such as Ebola, norovirus, respiratory syncytial virus, rhinovirus, and influenza^17^. A previous study concluded that FAV effectively inhibited tamoxifen-resistant breast cancer cells via targeting hTERT^18^. It is noteworthy that several antiviral medications have exhibited potential in both preventing and treating cancer^19^. The repurposing of antiviral agents for cancer treatment is grounded on that cancer patients, who frequently experience immunosuppression, are often administered antimicrobial agents concomitantly with chemotherapy for prophylaxis or therapy against various infectious conditions. Intriguingly, cancer patients who receive such combined therapeutic regimens have demonstrated increased survival rates and improved treatment outcomes compared to patients who receive chemotherapy alone^15^. Hence, antiviral agents can be used for combating tumor progression, thereby reducing the likelihood of infections and associated adverse effects.

On the flip side, one of the most efficacious approaches for targeted drug delivery entails the incorporation of the drug into a nanosized delivery system^20,21^. The growing demand for eco-friendly and sustainable products has driven the development of innovative “green” delivery systems. Green nanotechnology leverages the principles of green chemistry to prepare eco-friendly nanoparticles, reducing hazards to environment and the patients. The green nanotechnology approach is straightforward, cost-effective, environmentally friendly, free of organic solvents, and results in a non-toxic dosage form. We aimed to utilize the eco-friendly and cost-effective green nanoemulsion (NE) system developed in our previous research^22^. This is an incremental innovation where FAV was formulated into a nanocarrier delivery system without the usage of organic solvents. The use of safe and nontoxic oily phase Miglyol® 812 and low surfactants mixture concentration, will result in the formulation of “green” nanoemulsions without organic solvents that align with the principles of green chemistry and sustainability^23^. The nanoscale size of the NE, in addition to its added surfactants, namely Cremophor RH40® and Transcutol HP® could enhance penetration and significantly improve drug permeation^24,25^. This approach has the potential to target tumor cells actively or passively, thereby enhancing the therapeutic efficacy at the intended site, while concurrently mitigating both systemic toxicity and circumventing specific forms of multidrug resistance^20^. Additionally, adopting green principles contributes to the development of a straightforward and sustainable approach for drug delivery, while simultaneously reducing potential risks and hazards to patients and the environment^26^. Researchers are becoming more and more interested in environmentally friendly analytical procedures, especially in light of the health and environmental risks connected to organic solvents that are frequently utilized. The goal is to find new substitutes for dangerous chemicals, solvents, and reagents, or at the very least, to reduce their use to safe levels while preserving the methods’ efficacy.

The study introduces the use of three advanced tools to assess the greenness impact of the developed procedure^27–35^. AES provides assessment on the type and quantity of solvents employed, as well as the amount of waste produced during the analytical process. While AGREEprep uses 10 green sample preparation principles to assess how sample preparation techniques affect the environment. AGREEprep makes sure that even the first steps of the analytical process are ecologically friendly by concentrating on the preparation procedures, which improves the method’s overall sustainability. GAPI provides a thorough visual assessment of how the analytical process affects the environment. It evaluates every stage of the procedure, including the pre-analytical ones, giving a comprehensive picture of the ecological impact of the method.

These tools give a thorough rundown of how the method affects the environment, including everything from sample preparation to general procedure. The process is carefully examined for its environmental impact at every stage using a range of evaluation tools, resulting in a thorough green profile of the process. This work adds significantly to the field of sustainable chemistry and is in line with the global trend towards sustainable practices. Therefore, the present study focuses on further investigating the proposed role of FAV and the green favipiravir nanoemulsion (FNE) in selectively inducing cytotoxicity against breast cancer cells over normal cells while utilizing green RP-HPLC technologies.

## Results and discussion

### Characterization of favipiravir loaded nanoemulsion

#### Determination of particle size (PS), polydispersity index (PDI) and zeta potential (ZP)

When considering the critical factors that affect the interactions between the cells and the nanomaterials, we conclude that cellular uptake is greatly influenced by the size, size distribution, and surface charge of nano^36–38^. In our study, the NE system that was prepared exhibited a particle size of 25.29 ± 0.57 nm (Figure S1&S2). The mean nanosized droplets could be attributed to the utilization of an appropriate surfactant/co-surfactant mixture, which was adsorbed on the oil-water interface, thereby reducing the free energy of the system, and resulting in a small globule size^39^. The composition and ratio of the surfactant/co-surfactant mixture used in the current study were determined based on our prior research study^22^. The nano size of the NE is advantageous due to its large surface area to volume ratio, thus facilitating cellular penetration^40^ and avoiding macrophages engulfment^41^. Furthermore, the immune system is less likely to identify the NE as a foreign body due to its diminutive size^42,43^. The agglomeration of nanoparticles can significantly influence their interactions and subsequent cellular response, as agglomerated nanoparticles tend to have a larger size compared to individual ones. In our study, the PDI values of the FNE formulation ranged from 0.32 to 0.01, indicating a homogeneous and monodisperse system. Furthermore, the FNE formulation exhibited a zeta potential value of (-6.79 ± 5.52 mV) (Figure S3&S4). On one hand, cationic nanomaterials can establish strong interactions with cell membranes that have a negative charge, leading to rapid internalization, they also have the potential to cause membrane distortions^44^. On the other hand, anionic nanostructures can enter cells by crossing the negatively charged cell membrane with less harmful effects, as their net charge is similar to that of the cell membranes^45^. Additionally, phagocytic cells have been found to selectively uptake anionic nanoparticles^46^.

#### Transmission electron microscopy (TEM)

**Fig. 2:**
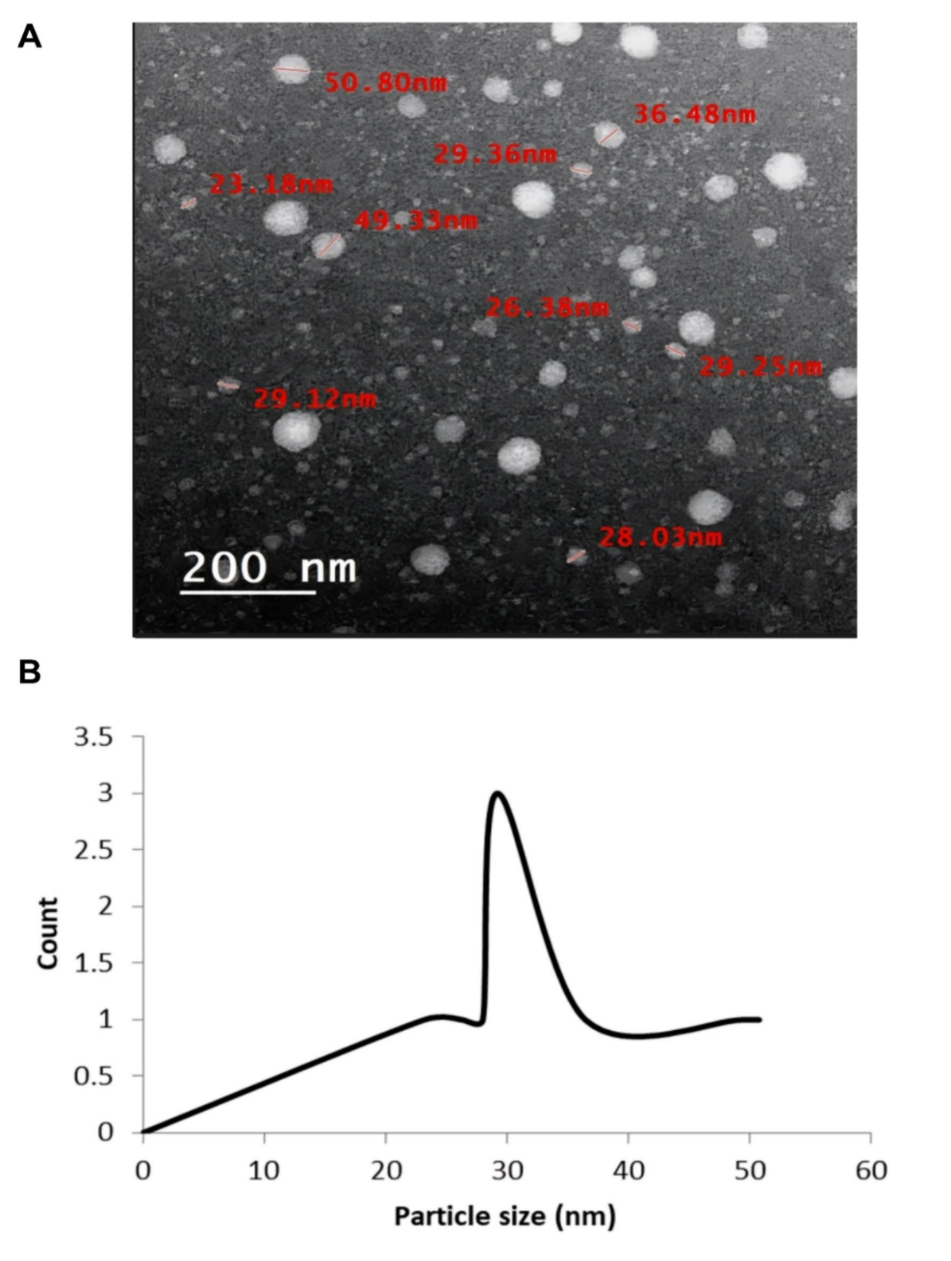
Transmission electron micrographs of FNE (**A**) and particle size distribution curve of TME of FNE (**B**).

The morphology of the FNE was characterized using TEM (JEM-2100, JEOL, USA). The photographs presented in Fig. 2a revealed that the FNE formulation consisted of spherical-shaped globules with a uniform surface. Furthermore, the particle size distribution graph Fig. 2b of the TEM image reveals that the FNE globule size was 29.24 nm^47–49^.,

#### Spectroscopic characterization of percentage transmission

**Fig. 3:**
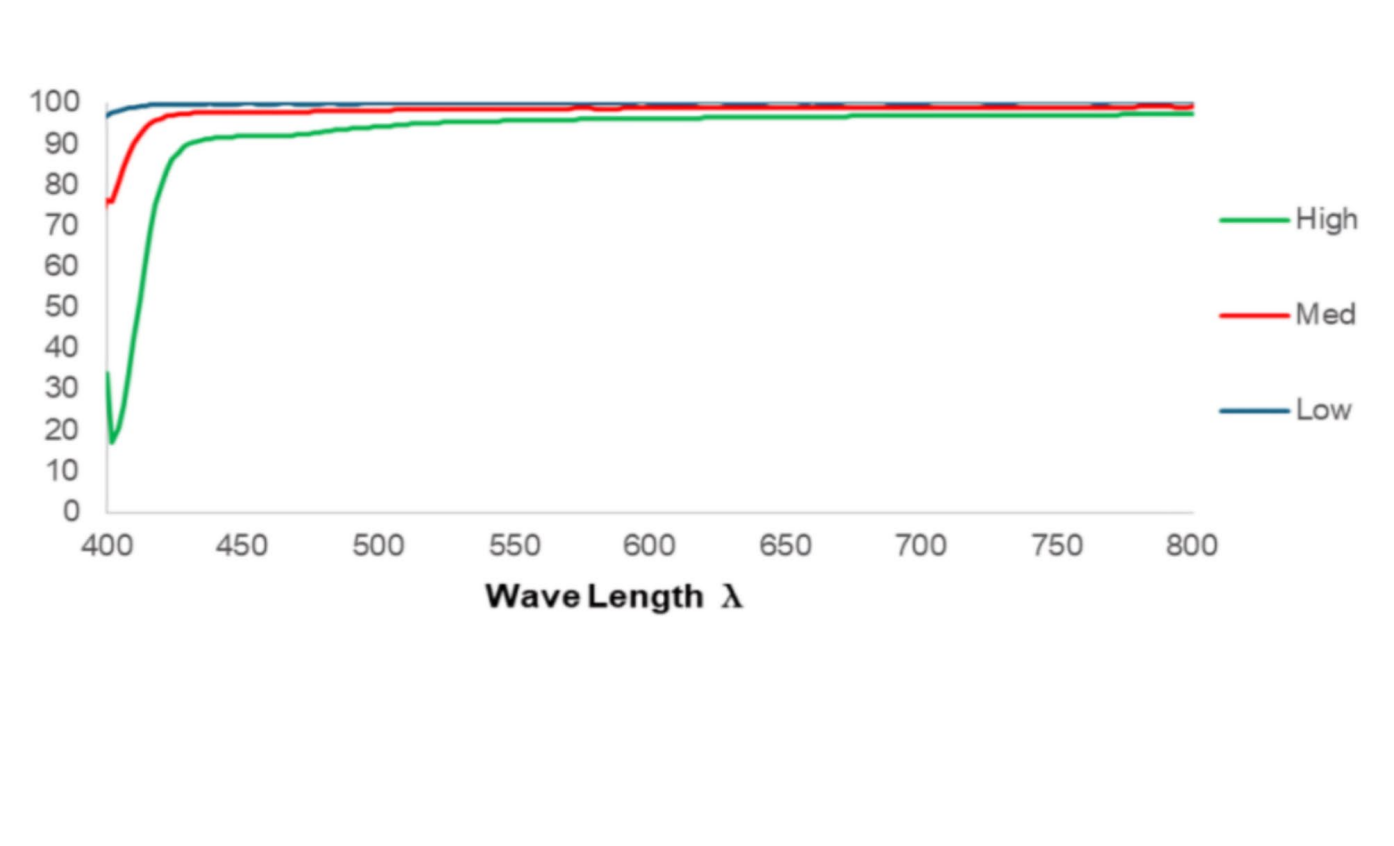
UV-Visible data of FNE.

The percentage transmission (%T) for the diluted FNE was shown in Fig. 3. A high FNE concentration (500 µl FNE/20 ml double-deionized water) exhibited a %T of 93.10%. Conversely, a medium concentration (50 µl FNE/20 ml deionized water) yielded a %T of 96.9%. Finally, a low concentration (5 µl FNE/20 ml double-deionized water) resulted in a %T of 99.6%, indicating increased successful nanoemulsion formulation with high degree of transparency. It was found that, a systematic decrease in FNE concentration resulted in a corresponding increase in transmittance (%T) within the UV-Vis spectral range (400–800 nm)^50^, which indicates an in direct relationship between FNE concentration and solution clarity. It was also found that the %T of all concentrations was above 90% secifically demonstrating a highly transparent solution. These findings are consistent with the previously observed fine oil droplet dispersions, evidenced by the small globule size^51,52^.

### Assessment of thermodynamic stability

The FNE formulation that was tested exhibited thermodynamic stability and demonstrated the ability to withstand a variety of stress tests, including centrifugation, heating-cooling, and freeze-thaw cycles, all without displaying any drug precipitation. These results are consistent with prior research conducted by the authors and with the work of Mahmoud, Al-Suwayeh et al., who utilized a high HLB surfactant and low HLB co-surfactant to successfully produce a stable NE. This suggests that the formulation is suitable for scaling up in an industrial setting, and has the potential for commercialization, especially when considering the use of different drugs with varying solubilities^53^.

### Cytotoxic effect of the FAV dispersion and FNE

**Fig. 4:**
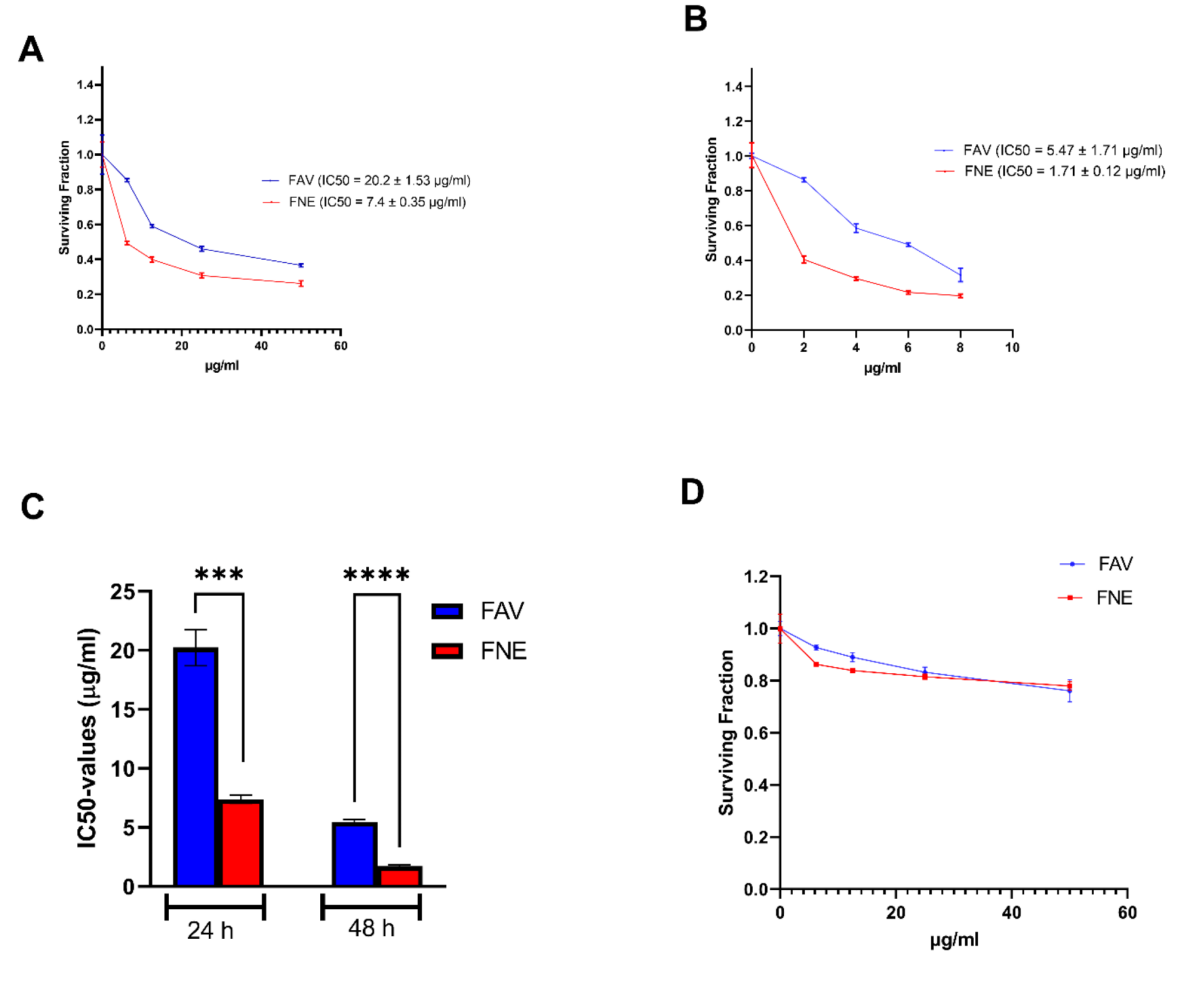
The effect of different concentrations of the FAV and FNE on MCF-7 breast cancer cell viability after 24 h (**A**) and 48 h (**B**) treatment, and their IC50 values (**C**) and their effect on Vero normal cells (**D**). The cell viability was assessed after 24–48 h by SRB assay. The data are presented as the means and standard deviations of triplicate observations from three independent experiments.

This assay was used to assess the growth inhibitory effect of the FAV dispersion and FNE on MCF-7 breast cancer after 24 and 48 h of treatment as shown in Fig. 4A and B, respectively. Moreover, the comparative IC50 values are shown in Fig. 4C. Both FAV and FNE produced a dose-dependent decrease in cell viability in MCF-7 cells. The FNE exhibited a more potent cytotoxic effect in MCF-7 cells with IC50 values of 7.4 ± 0.35 and 1.71 ± 0.12 µg/mL at 24 and 48 h, respectively, when compared with that of FAV with IC50 values of 20.2 ± 1.53 and 5.47 ± 1.71 µg/mL at 24 and 48 h, respectively. The formulated FNE significantly decreased IC50 values with 2.73- and 3.2- folds compared to the FAV dispersion at 24 and 48 h, respectively (*P* < 0.0001). These findings agreed with previous studies that reported the inhibitory effect of FAV on A549 lung cancer cells^54^. In addition, nanoemulsions showed major improvements in cancer therapy by being specific to escape from multi-drug resistance cancer cells and its efficiency in drug delivery targeting^55,56^. On the other hand, both FAV dispersion and FNE are almost nontoxic and didn’t show cytotoxic effect on VERO normal cells as shown in Fig. 4D. Likewise, nanoemulsions are one of the least toxic nano formulations due to its low surfactant mixture content and the fact that the specific oil (Miglyol 812) used in our nanoemulsion is generally regarded as safe chemical^57,58^.

### Cellular uptake

**Fig. 5:**
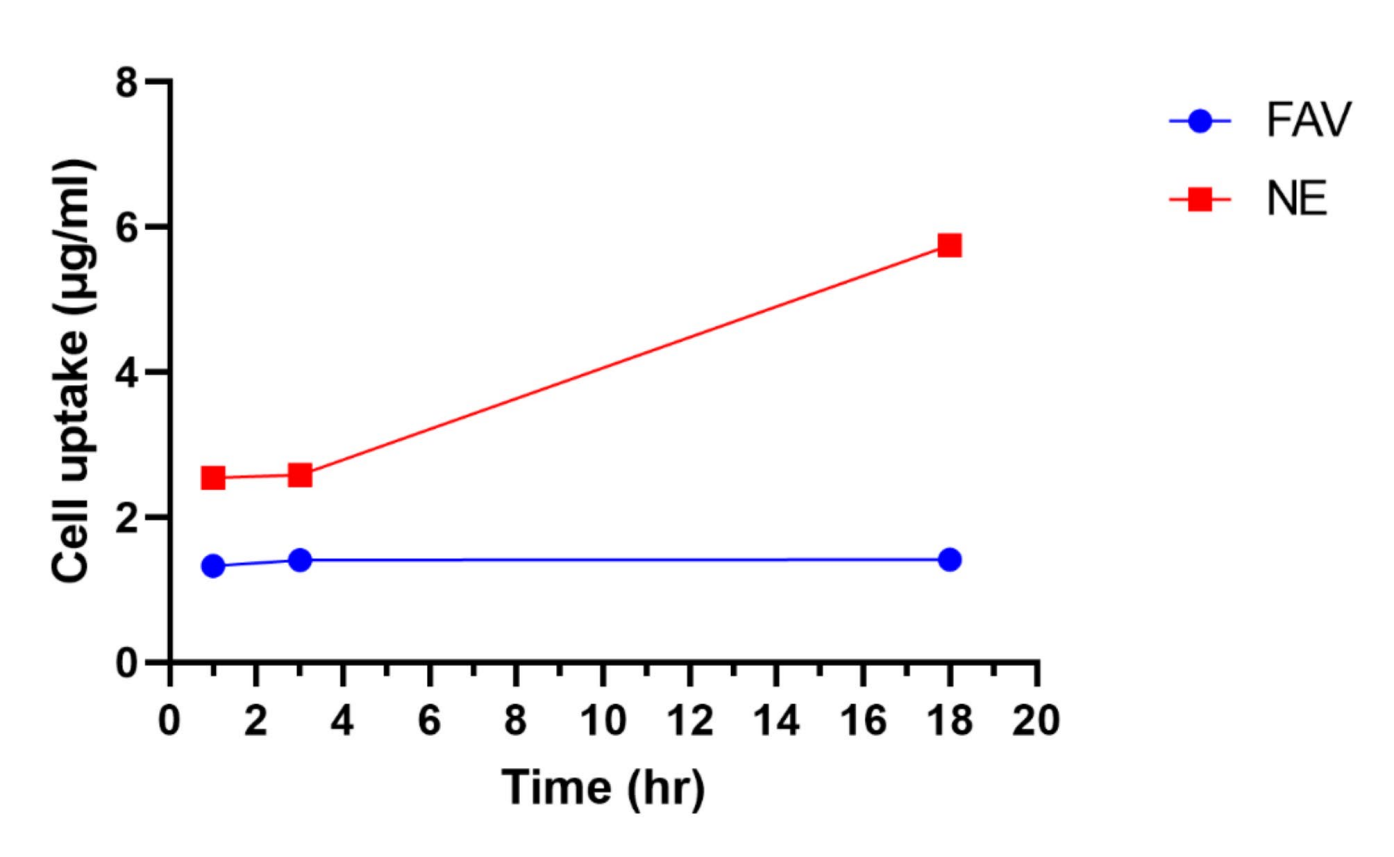
Invitro cellular uptake of Caco-2 cells. Cells were exposed to FAV and FNE for 1 h, 3 h and 18 h at 100 μm.

In order to assess the efficiency of cellular uptake in Caco-2 cells, the intracellular accumulation of FAV was analyzed using the HPLC method. The cellular uptake of FAV was compared between FNE and FAV dispersion. The findings revealed that the uptake of FAV from FNE by the cells was significantly higher after 1, 3, and 18 h compared to the uptake from FAV dispersion. In cells treated with FNE, the buildup of FAV uptake is time-dependent, increasing with time Fig. 5. Various studies have shown that the uptake of drugs from NE is greater when compared to the drug dispersion^59,60^. These findings suggest that FNE may allow for dose reduction while maintaining its therapeutic effect, hence decreasing drug’s toxicity and adverse effects.

### Greenness assessment tools

#### Analytical eco-scale tool

The evaluation of green chemistry processes frequently considers elements like the kind and volume of solvents utilized as well as the quantity of waste produced throughout the process. The Analytical Eco-Scale (AES) greenness evaluation tool was utilized in this investigation^61^.

It assigns a numerical score to a given method based on various factors such as chemical use, waste generation, energy consumption, and resource utilization. The AES tool finds applications in several areas; it can be utilized to optimize existing analytical methods by identifying areas of high environmental impact. By quantifying the greenness score, researchers can modify the method parameters, reagents, or procedures to reduce the overall environmental footprint while maintaining analytical performance.

It enables also the comparison of different analytical methods or variations within a method. Researchers can evaluate and select the most environmentally friendly option by considering the greenness scores of each method. This helps in making informed decisions regarding method selection based on sustainability criteria. When developing new analytical methods, it can guide researchers in designing environmentally friendly procedures from the outset. By considering the greenness score during method development, researchers can proactively incorporate green chemistry principles, optimize resource utilization, and minimize the environmental impact.

Using a mathematical formula, this tool calculates a procedure’s score in terms of penalty points. Subtracting the total number of penalty points from 100 yields a result that indicates the process’s environmental friendliness. A score of 75 or above indicates “excellent” performance, a score of 50 or more suggests “acceptable,” and a score of 50 or less indicates “inadequate.” These ratings are based on an environmental sustainability scale.

The penalty points and the results of the AES computations for the proposed technique are presented in Table 1. According to the AES evaluation, the approach received a score of 77, suggesting that it is an excellent green method.

**Table 1:** Summary penalty points for the method assessment.

Method assessment			
Studied factor	Description	Sub-total penalty point	Score total
Methanol	Quantity (2)	12	Σ = 23
	Hazardous (3 pictogram) Danger (2)		
Orthophosphoric acid	Quantity (2)	2	
	Hazardous (1 pictogram) Danger		
Diethylene glycol monoethyl ether	Quantity (2)	2	
	(1 pictogram)		
Caprylic/Capric triglyceride (Low amount)	Safe	0	
Polyoxyl 40 hydrogenated castor oil (Low amount)	Safe	0	
Water	Safe	0	
Occupational hazard	Safe	0	
Consuming instrumental energy	0.1 kWh per sample	1	
Waste	between (1–10) mL	3	
	No dealing	3	

Total score for the analytical eco-scale: 100 − 23 = 77 so the method is considered Excellent green.

#### Analytical greenness tool for sample preparation (AGREEprep)

First, AGREEprep, a sample preparation-focused measure, assesses the technical components of the process and pinpoints areas that could be used for improvement. It includes easy-to-use software that illustrates the efficacy of the approach visually. All analytical procedures must begin with sample preparation since it is necessary for analyte separation/enrichment, matrix removal/minimization, and technical compatibility. Despite being essential, sample preparation can have a negative environmental impact due to the large amounts of solvents, reagents, chemicals, materials, and energy needed. Because of this, the Green Analytical Chemistry (GAC) approach suggested that the first of its 12 principles—the total avoidance of this phase—be the benchmark for excluding sample preparation from green analytical processes. To bridge the gap in inclusion brought about by the GAC, Green Sample Preparation (GSP) was recently established into 10 principles to protect the environment and human health.

However, the analytical procedures that are completed prior to and subsequent to sample preparation are covered by the first and ninth principles, respectively. Most of these guidelines have a direct bearing on how samples are prepared. The 10 GSP principles do not assess the environmental impact of the sample, but they do provide a roadmap for the creation of more ecologically friendly analytical techniques. Because of this, AGREEprep, a brand-new measuring tool that is the first documented measure centered on sample preparation, has been suggested recently. Ten successive assessment sessions, which correlate to the 10 GSP principles, form the foundation of AGREEprep (Pena-Pereira et al. 2022). AGREEprep offered acceptable levels of information in comparison to other published measures since those metrics did not consider the sample preparation stage.

AGREEprep assessment not only evaluates greenness but also helps find technique strengths and weaknesses to improve the environmental friendliness of sample preparation procedures. The source code and the open access software for AGREEprep can be found at mostwiedzy.pl/AGREEprep, and git.pg.edu.pl/p174235/agreeprep, respectively.

In AGREEprep, scores for each of the 10 unique evaluation periods range from 0 to 1, with the highest and lowest achievement levels indicated by the peaks, respectively. The total score, which also ranges from 0 to 1, is obtained by adding the weighted scores of each criterion. A score of 1 indicates the best performance.

**Fig. 6:**
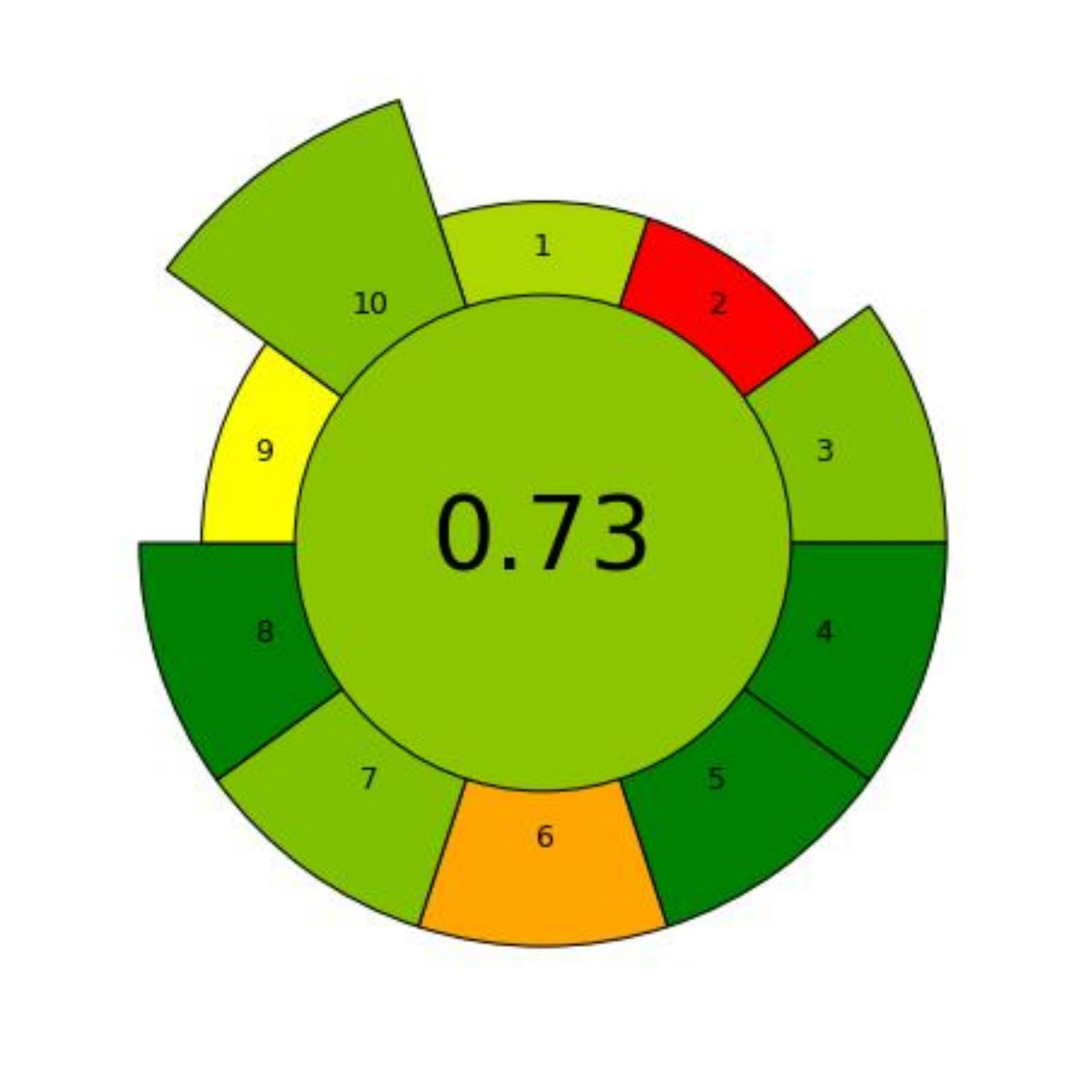
AGREEprep pictogram for the suggested method.

Based on the data presented in Fig. 6, the AGREEprep score of the proposed method is determined to be 0.73, indicating its environmentally friendly and sustainable nature.

#### Green analytical procedure index (GAPI)

By combining elements from Analytical Eco-Scale, a new instrument known as GAPI^30^ was created to offer thorough and qualitative data. The methodology’s overall environmental impact, including all stages from sample collection to final determination, is assessed by GAPI. The tool evaluates and measures the environmental impact related to each phase of the approach by using a particular symbol made up of five pentagrams. Three color codes are used by GAPI: red denotes a significant environmental risk, while yellow and green stand for reduced risk and greater greenness, respectively. This application gives users and readers access to comprehensive information on analyzed processes in an easily comprehensible manner.

**Fig. 7:**
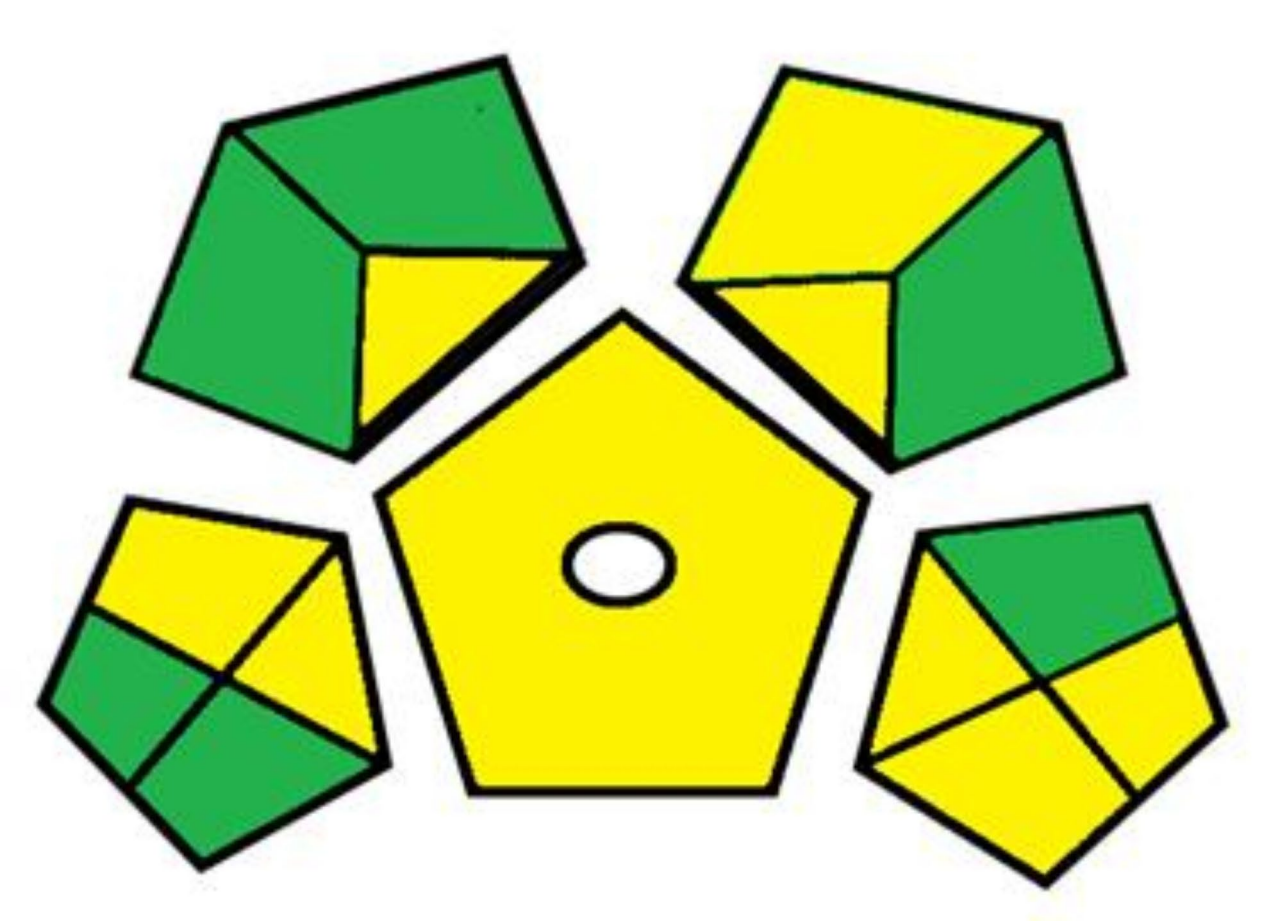
GAPI evaluation pictogram for the suggested HPLC method.

The GAPI index, which ranks the suggested approach for examining the drug, is shown in Fig. 7. There are no red pentagrams in the GAPI index of the suggested green technique; only six green and nine yellow pentagrams are present. According to the GAPI pentagrams, the suggested green approach is seen as ecologically beneficial since it employs greener solvents, which lower waste generation.

## Materials and methods

### Materials

#### Drugs and chemicals

FAV was obtained from Rameda Pharma (Cairo, Egypt). Transcutol® HP (diethylene glycol monoethyl ether) and Miglyol® 812 (caprylic/capric triglyceride) were kind gifts from Gattefosse (SaintPriest, Lyon, France) and EIPICO for pharmaceutical industries (Cairo, Egypt), respectively. Moreover, Cremophor® RH40 (polyoxyl 40 hydrogenated castor oil) was kindly provided by BASF SE (Ludwigsh, Germany). Methanol and ortho phosphoric acid HPLC grade were obtained from Sigma-Aldrich (Darmstadt, Germany). All other used chemicals and reagents were of analytical grade.

#### Cell lines

MCF-7 human breast cancer cell line, Caco-2 human colorectal adenocarcinoma cell line, and Vero normal cell lines were supplied by the American Type Culture Collection (ATCC, Manassas, MN, USA). To sustain the tumor cells, serial subculturing was performed at the National Cancer Institute in Cairo, Egypt using RPMI-1640 medium supplemented with 1% penicillin/streptomycin and 10% fetal bovine serum (FBS). Cells were subcultured to pre-confluence and incubated at 37 °C in a humidified environment with 5% CO_2_.

### Methods

#### Preparation of favipiravir-loaded nanoemulsion

**Fig. 8:**
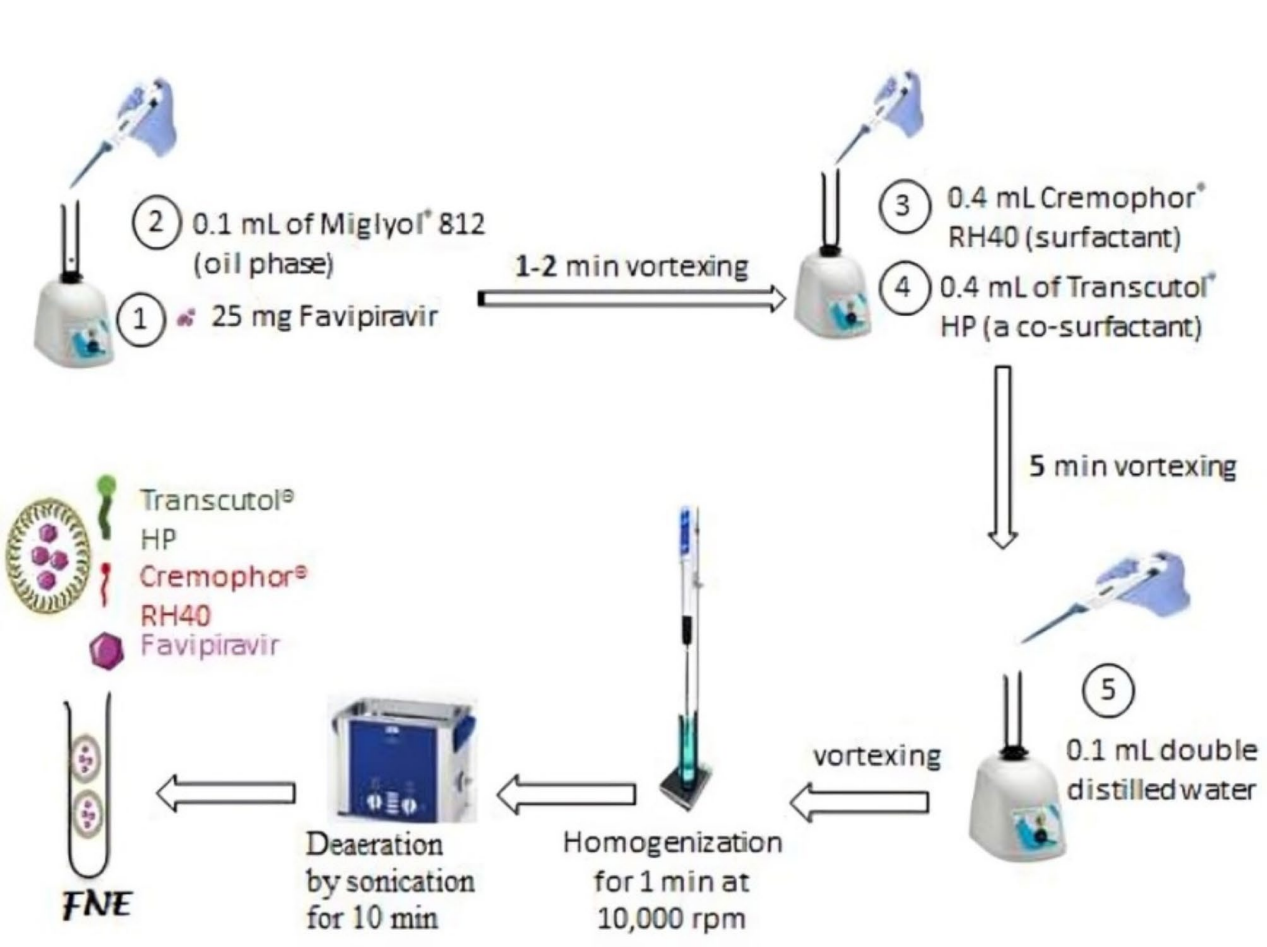
Schematic diagram for the Preparation of FNE.

FNE was synthesized employing the procedure outlined by Abd-Elrasheed et al.^22^ as shown in Fig. 8. In brief, a quantity of 25 mg of FAV was subjected to vortexing using a Vortex mixer (BenchMixer, Benchmark Scientific Inc., Edison, USA) along with 0.1 mL of Miglyol® 812, which served as the oil phase, for 1–2 min. Subsequently, 0.4 mL Cremophor® RH40 (surfactant) and 0.4 mL of Transcutol® HP (a co-surfactant) was introduced and vortexed for a duration of 5 min. The dropwise addition of 0.1 mL of double distilled water was carried out, vortexed, and finally, 1 min homogenization was performed at 10,000 rpm. Deaeration process was performed by the sonication (Ultrasonic Cleaner, Lab Companion UCP-10) of FNE for 10 min.

### Characterization of favipiravir-loaded nanoemulsion

#### Determination of particle size (PS), Polydispersity Index (PDI) and Zeta Potential (ZP)

The PS and PDI of FNE were evaluated in triplicate at 25 ± 1 °C using dynamic light scattering (DLS) analysis, conducted with the Malvern Zetasizer Nano ZS, Worcestershire, UK. For the analysis, a 0.1 mL aliquot of the developed FNE formulation was diluted with double-deionized water and mixed to ensure even dispersion. The fluctuations in the intensity of a scattered laser beam over time, resulting from the Brownian motion of droplets at an angle of 90º was measured.

The Doppler effect, a well-established phenomenon, is utilized in the technique of Laser Doppler electrophoresis to measure the motion of charged particles in an electric field. To perform this, a dispersion is placed in a cell containing two electrodes. Subsequently, an electric field is activated between these electrodes, causing particles with either a net charge or a net zeta potential to move towards oppositely charged electrode, exhibiting a specific velocity called mobility. This mobility is then mathematically transformed into the zeta potential of the charged particles.

#### Transmission electron microscopy (TEM)

Morphology of FNE was studied using JEOL, JEM 1230 TEM, Tokyo, Japan. 1–2 drops of the NE were suitably diluted (1:100) with double distilled water and applied on TEM grid (400- mesh carbon coated grids), then treated with a drop of 1% phosphotungistic acid and left for 30 s to negatively stain the sample, negative staining used to enhance the contrast and improve the taken images^62^. The coated grid was dried and then taken on a slide and observed under the microscope.

#### Spectroscopic characterization of percentage transmission

To assess the nanoemulsion formulation and transparency of FNE, three different concentrations were prepared. Serial dilutions of of 500 µL, 50 µL, 5 µL of FNE were dispersed each in 20 ml of double-deionized water. Following the preparation of these FNE samples, the percentage of light transmission through the formulations was measured across the broad spectral range of 200 to 800 nanometers. This comprehensive assessment of transparency was conducted to ensure the successful formulation of nanoemulsion with the desired level of clarity and light-transmitting properties of FNE system^52^.

#### Assessment of thermodynamic stability

FNE formulation was centrifuged at a speed of 3500 rpm for a duration of 30 min. Subsequently, the formulation was subjected to multiple cooling and heating cycles, with temperatures ranging from 4 °C to 45 °C. Additionally, the formulation was subjected to three freeze and thaw cycles at temperatures of -21 °C and + 25 °C, respectively, with each temperature being maintained for a minimum of 48 h. Finally, the formulation was observed for any signs of phase separation^63^.

#### Cytotoxic effect of the FAV dispersion and FNE

The cytotoxicity of FAV dispersion and FNE against MCF-7 breast cancer and VERO normal cells were investigated. The cell lines were plated at a density of 2 × 10^^3^ cells per well in 96-well microtiter plates containing RPMI-1640 media supplemented with FBS. After an incubation period of 24 h, MCF-7 cells were exposed to various concentrations of FAV or FNE for 24 h (0–60 µg/mL) and 48 h (0–10 µg/mL). VERO cells, on the other hand, were treated with FAV or FNE at concentrations ranging from 0 to 60 µg/mL for 48 h. Cytotoxicity of the remaining viable cells was evaluated using the sulphorhodamine-B (SRB) assay^64^. The optical density (OD) at 570 nm was measured using an ELISA microplate reader.

The surviving fraction was determined by dividing the OD of the treated cells by that of the control untreated cells. All experiments were performed in triplicate, and the results were presented as the mean ± standard deviation (SD). The software GraphPad Prism 8.4.2 was utilized to determine the 50% inhibitory concentration (IC50) from the dose-response curves.

#### Invitro Cellular Drug Uptake by HPLC and its chromatographic conditions

The test was conducted to evaluate the influence of FNE system on cellular uptake of FAV by Caco-2 cells. Caco-2 cells (2 × 10^6^ cells/well) were incubated in 6-well plates for 24 h. Then 15.7 µg/mL (100 µM) of either FAV dispersion or FNE was added to the culture media. Cell pellets were collected by trypsinization, dissolved in 0.5 mL methanol, sonicated for 15 min, and centrifuge at 4500 rpm for 5 min. The supernatant was then filtered over a syringe filter (0.22 μm) and measured by chromatographic system Shimadzu HPLC system (Columbia, MD, USA), isocratic pump and connected with Photodiode array detector. Chromeleon 7.1 edition software was used on the connected computer. Jasco Borwin version 1.5, LC-Net II/ADC system was used for data integration. For the standard preparation, a serial dilution of FAV (10–60 µg/mL) was prepared and then filtered on a syringe filter (0.22 μm). RP-HPLC was carried out at ambient temperature on Kromasil C_18_ (5 μm, 250 mm x 4.6 mm I.D) column. The filtered and degassed mobile phase consisted of 0.1% orthophosphoric acid in 1 L water and methanol (80%:20%) in an isocratic mode. A 0.22 μm syringe filter was used to filter the mobile phase and was then delivered at a flow rate of 1.5 mL/min. The injection volume was 10 µL. PDA detection was achieved at 320 nm.

#### The assessment of the greenness

The green chemistry entails a variety of factors that necessitate a meticulous examination of each stage within an analytical procedure. There are different tools available for gauging the environmental impact of analytical methods, each with its own advantages and disadvantages. It is crucial to employ tools that encompass various aspects of green chemistry in order to conduct a comprehensive assessment.

In this study, a comprehensive evaluation of the environmental impact of the components used in nanoemulsion formulation and analytical procedure is carried out using a “green triple-tool” approach. The first tool, AES, involves the type, quantity of the used solvents and the entire process. The second tool, known as AGREEprep, evaluates the preparation steps based on 10 principles of green sample preparation. The third tool, GAPI, encompasses every stage of the procedure, including the pre-analytical steps, to provide a comprehensive overview of the method’s ecological impact. By employing this triple-tool approach, a holistic understanding of the method’s environmental sustainability is attained.

## Conclusion

Green FNE containing Cremophor® RH40 (40%), Transcutol® HP (40%), Miglyol® 812 (10%) and water (10%) was successfully prepared in the nano-size (25.29 ± 0.57 nm) with a zeta-potential of (-6.79 ± 5.52 mV) and %T of (91.25 ± 0.60), exhibited thermodynamic stability. This suggests that the formulation is easily scalable in an industrial setting. Our research findings indicate that both the proposed cytotoxicity of FAV in MCF-7 cells and cellular uptake were enhanced by incorporating FAV into the non-toxic green NE formulation that was prepared based on previous studies, leading to dose reduction and minimized adverse effects. This study used three evaluation tools; AES, GAPI, and AGREEprep to determine the greenness profile. These tools provided optical and numerical results to interpret the environmental impact of the methods. Integrating these tools ensured that every facet of the analytical process is a green, eco-friendly method since it depended on a greener solvent that resulted in diminished waste production. This work will be valuable and supportive for users working on a straightforward, cost-effective, environmentally friendly and easily scalable nanoemulsion system. Moreover, this is complemented with the level of greenness of the developnd method where all greenness principles can be applied to minimize environmental impact and it can encourage new strategies to decrease the weakest points in given procedures.

### Future perspectives

Further studies are needed to conduct a comprehensive long-term stability study to further assess the robustness of our formulations. This will help understanding of the formula’s shelf life and ensure its efficacy over extended period. Additionally, we aim to explore the therapeutic potential of our new formula through rigorous testing in animal models to evaluate the safety and efficacy of the treatment. Moreover, we aim to detect the molecular pathways involved in the drug’s action against cancer using advanced bioinformatics tools. Throughout these future endeavors, we will prioritize sustainability by using green, economic, and efficient solvents in our formulations.

## Electronic supplementary material

Below is the link to the electronic supplementary material.

Supplementary Material 1

## Data Availability

The datasets generated during and/or analyzed during the current study are available in the supplementary material file submitted and from the corresponding author on reasonable request.
