# Supplementary material for: Innovative anti-proliferative effect of the antiviral favipiravir against MCF-7 breast cancer cells using green nanoemulsion and eco-friendly assessment tools
Source: Sci Rep. 2024 Nov 14;14:27939. doi: 10.1038/s41598-024-78422-2 (PMC11561084; doi:10.1038/s41598-024-78422-2)
Supplement: Supplementary file 1 — Supplementary Material 1 [file 41598_2024_78422_MOESM1_ESM.pdf]

Innovative Anti-proliferative Effect of the Antiviral Favipiravir against MCF-7 Breast Cancer Cells Using Green Nanoemulsion and Eco-Friendly Assessment Tools

Eman Abd-Elrasheed[1], Sally A. Fahim[2]*, Christine K. Nessim[3], Sara Nageeb El-Helaly[4]

[1] Department of Pharmaceutics and Industrial pharmacy, Pharmacy Program, St. Petersburg University, Egypt

[2] Department of Biochemistry, School of Pharmacy, Newgiza University (NGU), Newgiza, km 22 Cairo-Alexandria Desert Road, Giza, 12577; Egypt

[3] Department of Pharmaceutical Chemistry, Faculty of Pharmacy, Ahram Canadian University, 6th October City, Cairo, Egypt

[4] Department of Pharmaceutics and Industrial Pharmacy, Faculty of Pharmacy, Cairo University, Cairo, Egypt

*** Corresponding author:**

Tel: +20 1220909909; e-mail: sally.atef@ngu.edu.eg; sallyatef@hotmail.com, ORCID ID: 0000-0002-7934-5030

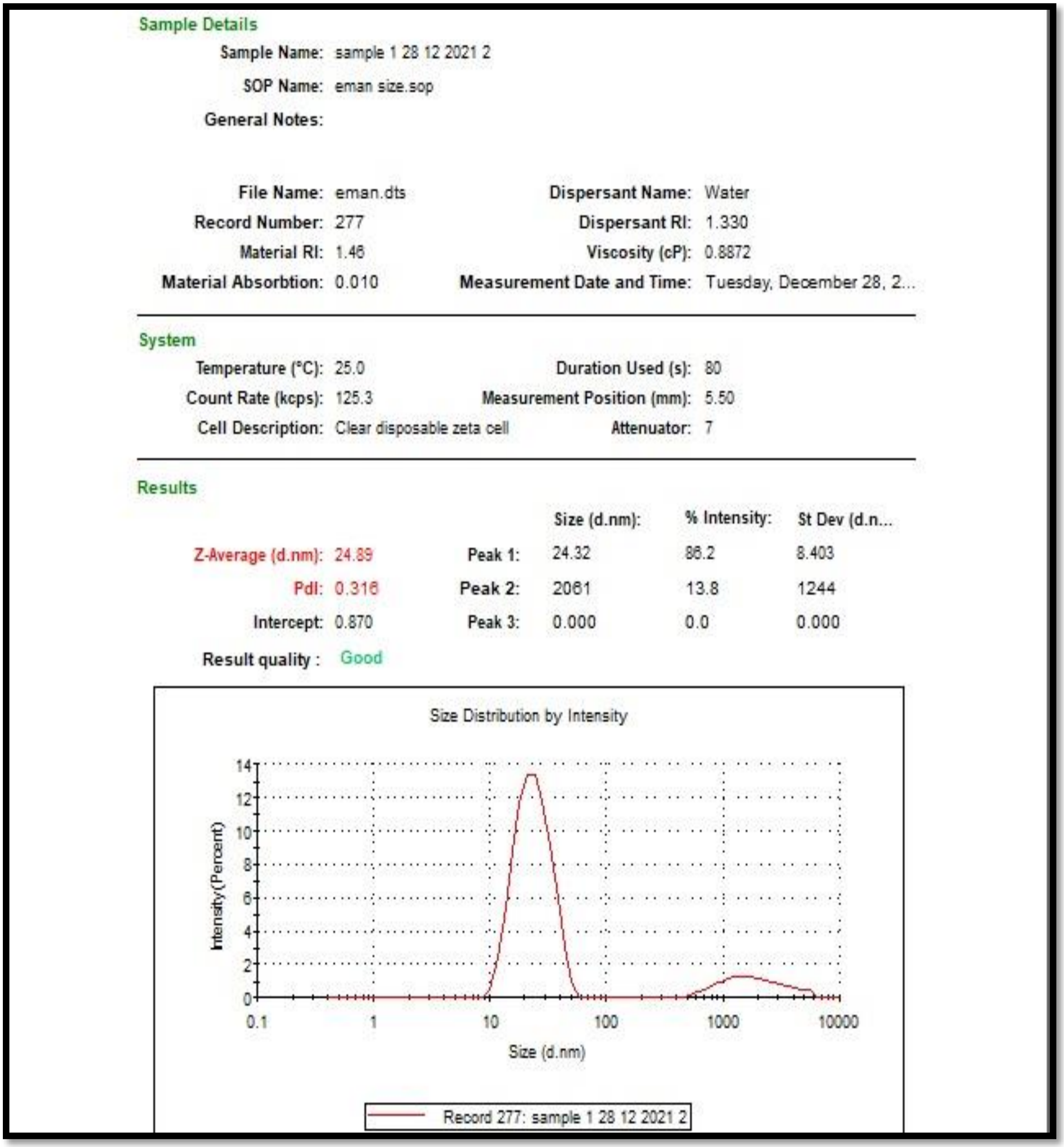

Sample Details

Sample Name:	sample 1 28 12 2021 2
SOP Name:	eman size.sop
General Notes:	

File Name:	eman.dts	Dispersant Name:	Water
Record Number:	277	Dispersant RI:	1.330
Material RI:	1.46	Viscosity (cP):	0.8872
Material Absorbtion:	0.010	Measurement Date and Time:	Tuesday, December 28, 2...

System

Temperature (°C):	25.0	Duration Used (s):	80
Count Rate (kcps):	125.3	Measurement Position (mm):	5.50
Cell Description:	Clear disposable zeta cell	Attenuator:	7

Results

			Size (d.nm):	% Intensity:	St Dev (d.n...
Z-Average (d.nm): 24.89		Peak 1:	24.32	86.2	8.403
PdI: 0.316		Peak 2:	2061	13.8	1244
Intercept: 0.870		Peak 3:	0.000	0.0	0.000
Result quality : Good					

Figure S1. Z-average particle size (PS) and polydispersity index (PDI) of FNE via (Malvern Zetasizer Nano ZS; Worcestershire, UK).

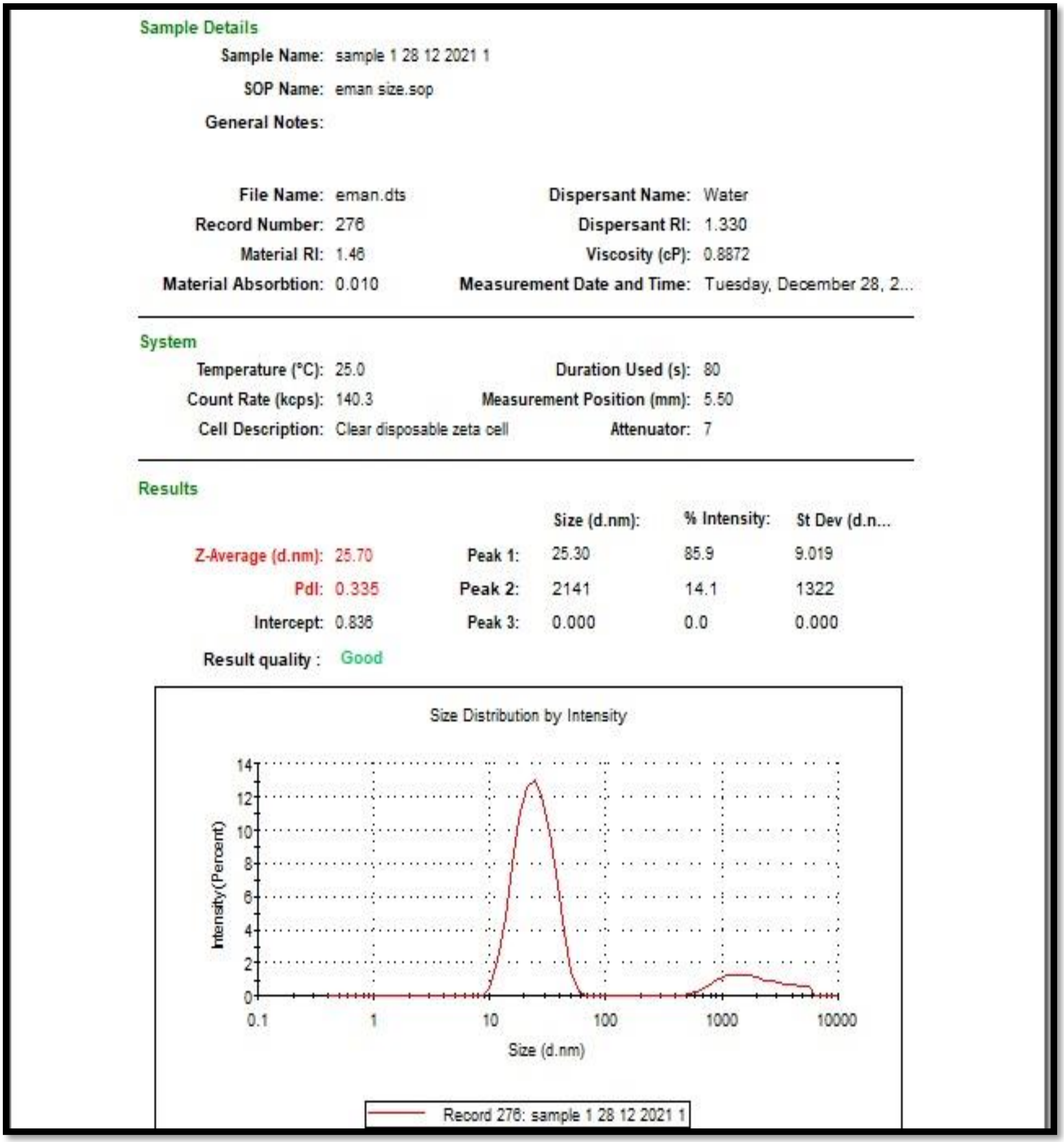

Sample Details

Sample Name: sample 1 28 12 2021 1

SOP Name: eman size.sop

General Notes:

File Name:	eman.dts	Dispersant Name:	Water
Record Number:	276	Dispersant RI:	1.330
Material RI:	1.46	Viscosity (cP):	0.8872
Material Absorbtion:	0.010	Measurement Date and Time:	Tuesday, December 28, 2...

System

Temperature (°C):	25.0	Duration Used (s):	80
Count Rate (kcps):	140.3	Measurement Position (mm):	5.50
Cell Description:	Clear disposable zeta cell	Attenuator:	7

Results

			Size (d.nm):	% Intensity:	St Dev (d.n...
Z-Average (d.nm):	25.70	Peak 1:	25.30	85.9	9.019
PdI:	0.335	Peak 2:	2141	14.1	1322
Intercept:	0.836	Peak 3:	0.000	0.0	0.000

Result quality : Good

Figure S2. Z-average particle size (PS) and polydispersity index (PDI) of FNE via (Malvern Zetasizer Nano ZS; Worcestershire, UK).

Sample Details

Sample Name: sample 1 28 12 2021 1

SOP Name: eman potential.sop

General Notes:

File Name:	eman.dts	Dispersant Name:	Water
Record Number:	278	Dispersant RI:	1.330
Date and Time:	Tuesday, December 28, 2021...	Viscosity (cP):	0.8872
		Dispersant Dielectric Constant:	78.5

System

Temperature (°C):	24.9	Zeta Runs:	24
Count Rate (kcps):	128.7	Measurement Position (mm):	2.00
Cell Description:	Clear disposable zeta cell	Attenuator:	9

Results

			Mean (mV)	Area (%)	St Dev (mV)
Zeta Potential (mV):	-2.89	Peak 1:	-2.89	100.0	4.14
Zeta Deviation (mV):	4.14	Peak 2:	0.00	0.0	0.00
Conductivity (mS/cm):	0.0457	Peak 3:	0.00	0.0	0.00
Result quality :	Good				

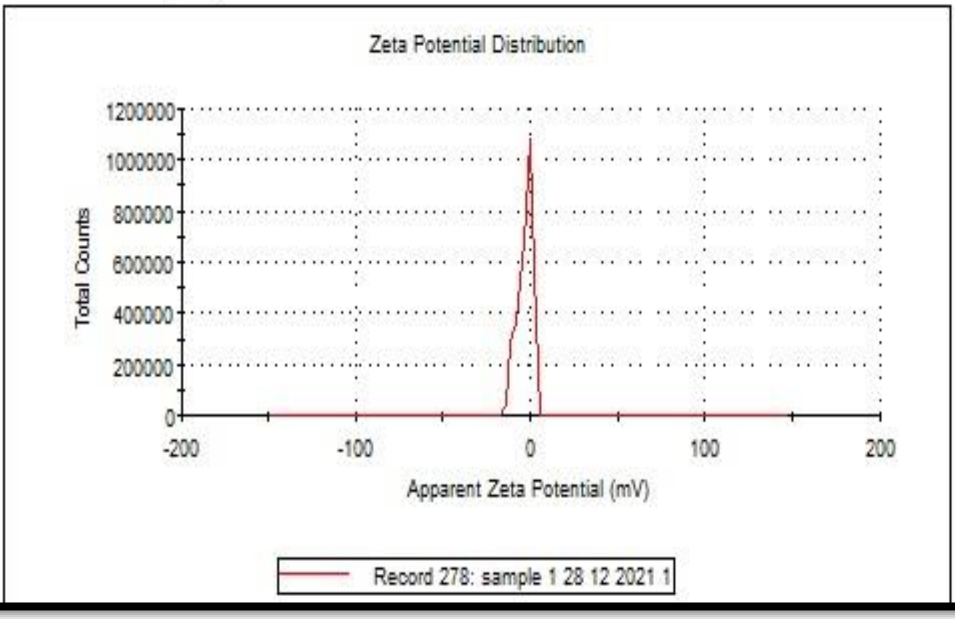

Figure S3. Zeta potential of FNE via (Malvern Zetasizer Nano ZS; Worcestershire, UK).

Sample Details

Sample Name:	sample 1 28 12 2021 2		
SOP Name:	eman potential.sop		
General Notes:			
File Name:	eman.dts	Dispersant Name:	Water
Record Number:	279	Dispersant RI:	1.330
Date and Time:	Tuesday, December 28, 2021...	Viscosity (cP):	0.8872
		Dispersant Dielectric Constant:	78.5

System

Temperature (°C):	24.9	Zeta Runs:	12
Count Rate (kcps):	163.4	Measurement Position (mm):	2.00
Cell Description:	Clear disposable zeta cell	Attenuator:	9

Results

			Mean (mV)	Area (%)	St Dev (mV)
Zeta Potential (mV):	-10.7	Peak 1:	-10.7	100.0	4.19
Zeta Deviation (mV):	4.19	Peak 2:	0.00	0.0	0.00
Conductivity (mS/cm):	0.0379	Peak 3:	0.00	0.0	0.00
Result quality :	Good				

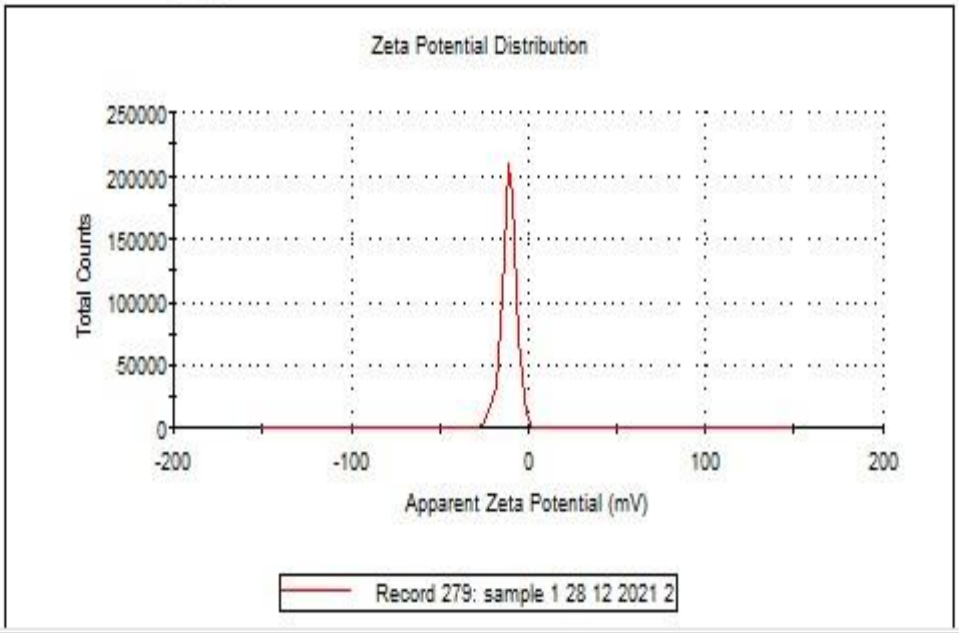

Figure S4. Zeta potential of FNE via (Malvern Zetasizer Nano ZS; Worcestershire, UK).
